# Pathological complete response and survival according to the level of HER-2 amplification after trastuzumab-based neoadjuvant therapy for breast cancer

**DOI:** 10.1038/sj.bjc.6605939

**Published:** 2010-10-26

**Authors:** S Guiu, M Gauthier, B Coudert, F Bonnetain, L Favier, S Ladoire, H Tixier, B Guiu, F Penault-Llorca, F Ettore, P Fumoleau, L Arnould

**Affiliations:** 1 Department of Oncology, Georges-François Leclerc Cancer Center, F-21000 Dijon, France; 2 Biostatistics Unit, Georges-François Leclerc Cancer Center, F-21000 Dijon, France; 3 EA 4184 School of Medicine, F-21000 Dijon, France; 4 Department of Surgery, Georges-François Leclerc Cancer Center, F-21000 Dijon, France; 5 Department of Radiology, CHU (University Hospital), F-21079 Dijon, France; 6 Department of Pathology, Jean Perrin Cancer Center, F-63011 Clermont-Ferrand, France; 7 Department of Pathology, Antoine-Lacassagne Cancer Center, F-06189 Nice, France; 8 Department of Pathology, Georges-François Leclerc Cancer Center, F-21000 Dijon, France

**Keywords:** pathological response, survival, fluorescence *in situ* hybridisation, HER-2 level amplification, trastuzumab

## Abstract

**Background:**

We analysed whether the level of human epidermal growth factor receptor-2 (HER-2) amplification significantly influenced either pathological complete response (pCR) or recurrence-free survival (RFS) and overall survival (OS) after trastuzumab-based neoadjuvant therapy.

**Methods:**

In all, 99 patients with an HER-2-amplified breast tumour treated with trastuzumab-based neoadjuvant therapy were included. Tumours were classified as low amplified (LA; 6–10 signals per nuclei) or highly amplified (HA; >10 signals). Pathological response was assessed according to Chevallier's classification (pCR was defined as grade 1 or 2). Median follow-up lasted 46 months (6–83). Cox uni- and multivariate analyses were performed.

**Results:**

In all, 33 tumour samples were LA and 66 were HA. The pCR in HA tumours was significantly higher than in LA tumours (55% *vs* 24%, *P*=0.005), whereas no association was found between the pCR rate and tumour stage, grade or hormone receptor status. In multivariate analysis, the pathological nodal status (*P*=0.005) and adjuvant trastuzumab (*P*=0.037) were independently associated with RFS, whereas the level of HER-2 amplification nearly reached statistical significance (*P*=0.057). There was no significant difference between LA and HA tumours for OS (*P*=0.22, log-rank).

**Conclusion:**

The level of *HER-2* gene amplification significantly influenced pCR but not RFS or OS in non-metastatic breast cancer treated with trastuzumab-based neoadjuvant therapy. However, RFS in patients with HA tumours tended to be shorter.

The human epidermal growth factor receptor-2 (*HER-2*) gene is amplified in 10–26% of human breast cancers (Gown *et al*, 2008). *HER-2* gene amplification is associated with the over-expression of the HER-2 protein in >95% of cases (Wolff *et al*, 2007). Both HER-2 over-expression and *HER-2* gene amplification have been correlated with poor clinical outcome (Slamon *et al*, 1987; Kallioniemi *et al*, 1991; Press *et al*, 1997). The HER-2 status is also a strong predictor of a clinical benefit from HER-2-targeted therapy, such trastuzumab (Herceptin; Roche, Neuilly-sur-Seine, France), a humanised monoclonal antibody directed against the external domain of HER-2 protein (Yamauchi *et al*, 2001). Several randomised trials have proved the efficacy of trastuzumab in metastatic (Slamon *et al*, 2001; Marty *et al*, 2005) and adjuvant (Piccart-Gebhart *et al*, 2005; Romond *et al*, 2005; Joensuu *et al*, 2006; Smith *et al*, 2007) settings for HER-2-positive breast cancer in terms of response rate, recurrence rate and a decrease in mortality. In the neoadjuvant setting, trastuzumab in association with chemotherapy had also shown a clinical benefit in terms of pathological complete response (pCR; Van Pelt *et al*, 2003; Buzdar *et al*, 2005; Coudert *et al*, 2006, 2007; Gianni *et al*, 2007).

American Society of Clinical Oncology/College of American Pathologists (ASCO/CAP) guidelines recommend using either immunohistochemistry (IHC) assays for initial evaluation of HER-2 status followed by reflex testing by fluorescence *in situ* hybridisation (FISH) of some IHC categories or FISH in initial testing (Wolff *et al*, 2007). The level of over-expression of HER-2 protein with IHC assays is a known predictive factor of response to trastuzumab (Slamon *et al*, 2001) and we have previously shown a positive correlation between the level of HER-2 amplification assessed by FISH and the rate of pCR to trastuzumab-based neoadjuvant treatment (Arnould *et al*, 2007). However, the relationship between the level of HER-2 amplification and the outcome of patients given neoadjuvant trastuzumab remains unclear.

The aim of this study was to determine whether the level of *HER-2* gene amplification using FISH assays significantly influenced recurrence-free survival (RFS) and overall survival (OS) in non-metastatic breast cancer treated with trastuzumab-based neoadjuvant therapy.

## Materials and methods

### Patients

Breast biopsies from 116 patients, who had received neoadjuvant trastuzumab in combination with chemotherapy for locally HER-2-positive breast cancer were retrospectively collected from 19 centres in France. All of the patients provided written, informed consent for their tissue material and clinical data to be centrally collected and used for research purposes. This study was approved by our institutional review board.

The patients were aged from 26 to 76 years (mean, 46.6 years) and had histologically confirmed, unilateral, unicentric, non-metastatic, HER-2-positive (in IHC) invasive ductal breast carcinoma. Most of the patients were treated in the framework of two open-label phase II clinical trials: GETN(A)-1 (*n*=63; Coudert *et al*, 2007) and TAXHER-S01 (*n*=21; Coudert *et al*, 2006). The remaining patients (*n*=32) had an equivalent preoperative regimen to that used in the TAXHER-S01 trial.

The 63 patients included in the GETN(A)-1 trial had received weekly neoadjuvant trastuzumab (4 mg kg^−1^ loading dose followed by 2 mg kg^−1^) in combination with docetaxel (75 mg m^−2^) and carboplatin (area under the curve of six) every 3 weeks for six cycles. Adjuvant trastuzumab was also administered in responding patients. The 21 patients included in the TAXHER-S01 trial had received the same preoperative schedule of trastuzumab in association with docetaxel (100 mg m^−2^) every 3 weeks for six cycles, but no adjuvant trastuzumab was scheduled in this study. Additional patients (*n*=*32*) received trastuzumab every 3 weeks (8 mg kg^−1^ loading dose followed by 6 mg kg^−1^) instead of weekly trastuzumab (Leyland-Jones *et al*, 2003) and adjuvant trastuzumab was also administered.

In all patients, 3 weeks after the last administration of neoadjuvant trastuzumab, tumours were surgically removed and pCR was assessed according to Chevalliers’classification: pCR was defined as no evidence of carcinoma either in the breast or in the lymph nodes, without (grade 1) or with (grade 2) *in situ* carcinoma. In accordance with institutional practices, adjuvant hormone therapy in patients with hormone receptor-positive tumours and adjuvant radiotherapy were mandatory.

### HER-2 status

The 84 patients included in the GETN(A)-1 or TAXHER-S01 trials were initially tested IHC 3+ or 2+ for HER-2 status. For all of the patients in this study, HER-2 status was re-analyzed centrally using both IHC and FISH assays by an experienced pathologist who was blinded to patient information, including the original IHC test results.

HER-2 status in IHC was evaluated with A485 polyclonal antibody (Dako, Glostrup, Denmark) or 4B5 monoclonal antibody (Ventana Medical Systems Inc., Tucson, AZ, USA) on the BenchMark XT system (Ventana Medical Systems Inc.): biopsies were graded according to the HercepTest (Dako) scoring system (0+, 1+, 2+, or 3+).

FISH analyses were carried out using the HER-2 Probe (Oncor, Gaithersburg, MD, USA) and BenchMark XT system. For each biopsy, HER-2 signals were counted in ⩾60 tumour cell nuclei and the mean HER-2 signals per nuclei was calculated. The level of HER-2 amplification in tumours was classified as follows: no amplified (NA; mean, <6 signals per nuclei), low amplified (LA; mean, 6–10 signals per nuclei), or highly amplified (HA; mean, >10 signals per nuclei or uncountable because of clusters of signals). The cutoff of 10 gene copies per nuclei to distinguish between LA and HA was chosen because it is the same as that proposed with chromogenic *in situ* hybridisation and also because above this cutoff, it is almost impossible to count signals precisely because of clusters and small aggregates. Borderline tumours (mean between four and eight signals per nuclei) were analysed by double-color FISH using a *HER-2*-gene-specific probe and a centromeric probe for chromosome 17 (PathVysion HER-2 DNA Probe kit, Vysis-Abbott, Abbott Park, IL, USA) to determine HER-2 amplification. In these cases, HER-2 amplification was defined by a ratio of HER-2 to chromosome 17 centromeric signals (HER-2/CEP17) of ⩾2.2 (Wolff *et al*, 2007). All the tumours with ⩾6 HER-2 signals per nuclei had a HER-2/CEP17 ratio ⩾2.2 and therefore, were amplified tumours. All the tumours with <6 HER-2 signals per nuclei had a HER-2/CEP17 ratio ⩽1.8 and thus, were NA tumours.

**Figure 1:**
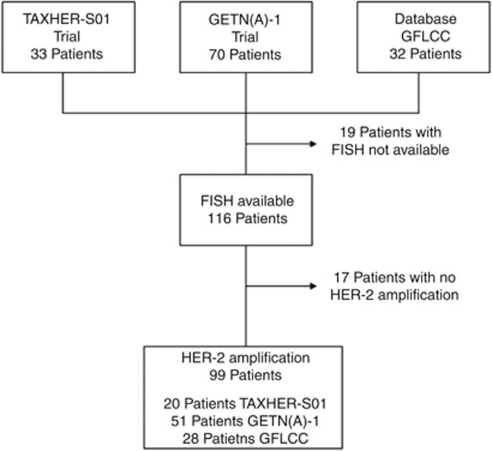
Flow chart of the study. Abbreviations: GFCLCC, Georges–François Leclerc Cancer Center database; FISH, fluorescence *in situ* hybridisation

Only patients with centrally confirmed HER-2 amplification were finally included in this study to evaluate pathological response rate (Figure 1). For RFS and OSs, NA tumours were also included.

### Statistical analysis

Qualitative variables were described using frequency and percentages. *χ*^*2*^ and Fisher's exact tests were used to compare patient or tumour characteristics according to the level of HER-2 amplification with FISH assays (NA, LA, and HA). For these analyses, Bonferroni adjustments were carried out to prevent inflation of type one error (the significant level was 0.016 for 3 comparisons).

Associations between tumour size, tumour grade, hormone–receptor status, level of HER-2 amplification, and the presence or absence of pCR were evaluated using univariate and multivariate logistic regression. To take into account the trial effect (GETN(A)-1, TAXHER-S01, and GFLCC database), analyses were adjusted for this factor.

The median follow-up was calculated using the reverse Kaplan–Meier method. Recurrence-free survival was defined as the time from the date of histology to the date of the first recurrence of breast cancer at any site or death from any cause. Surviving patients without recurrence were censored at the last follow-up. The OS was defined as the time from the date of histology to death from any cause. Survival distributions were estimated with the Kaplan–Meier method and compared using the log-rank statistic. Univariate (RFS and OS) and multivariate (RFS) Cox proportional hazards models stratified on the trial were fitted to test for an association between classical prognostic variables, the level of HER-2 amplification, pCR, adjuvant trastuzumab, and RFS or OS. Given the small number of events, multivariate analysis for OS was not performed. Akaike information criterion was computed for the goodness of fit for multivariate models and Harrell's *C*-statistic for discrimination (a Harrell's *C*-index=0.5 indicates no predictive discrimination and a Harrell's *C*-index=1.0 indicates perfect separation of patients) for each variable and for final multivariate Cox models. The multivariate models were internally validated using bootstrapping (100 replications). *P*-values were two-tailed and considered significant when less than 0.05. All analyses were performed using Stata V11 software (StataCorp LP, College Station, TX, USA).

## Results

### Patients and tumours

Baseline patient and tumour characteristics are summarised in Table 1. In all, 99 (85%) tumours were considered amplified after central FISH analyses, among which 33 were classified as LA tumours and 66 HA tumours. There were no significant differences between these two groups in terms of tumour stage, nodal status, hormone receptor status, or treatment given. The HA tumours had a higher histological grade than the LA tumours (*P*=0.01).

### Analysis of pCR

According to Chevallier's classification (Table 2), 44 (44.5%) patients had a pCR, whereas 55 (55.5%) had no or only a partial response. In univariate logistic analysis, only the level of HER-2 amplification (FISH) was related to pCR (*P*=0.005). In multivariate analysis, this variable was independently associated with pCR (*P*=0.024), whatever the trial (*P*=0.632).

### Recurrence-free survival according to HER-2 amplification

Median follow-up was 46-months (range, 6–83 months). Local or regional recurrences occurred in six patients (two HA tumours, two LA tumours, two NA tumours); one of these had a pCR. Metastatic recurrence (alone or with locoregional recurrence) occurred in 18 patients (14 HA tumours, 2 LA tumours, 2 NA tumours) among whom 5 had an initial pCR. Three patients died without a diagnosis of recurrent disease.

**Figure 2:**
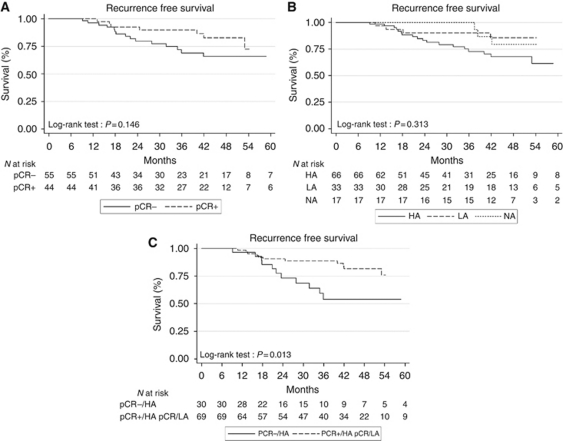
Recurrence-free survival according to the pathological response (**A**), the HER-2 copy number (**B**) and both pathological response and level of HER-2 amplification (**C**; Kaplan–Meier estimate). Abbreviations: HA, highly amplified tumours; LA, low-amplified tumours; NA, no amplified tumours; pCR+, pathological complete response; pCR−, absence of pathologiccal complete response.

RFS was not statistically different according to the pCR rate (*P*=0.145, log-rank test; Figure 2A), or according to the HER-2 copy number (*P*=0.313, log-rank test; Figure 2B) or the level of HER-2 amplification (*P*=0.161, log-rank test). In the univariate Cox analysis, the level of HER-2 amplification was not significantly associated with RFS (Table 3), whereas the pathological nodal status (HR=3.247 (CI 95%, 1.396–7.552), *P*=0.006) and adjuvant trastuzumab treatment (HR=0.157 (CI 95%, 0.045–0.539), *P*=0.003) were. In multivariate analysis, pathological nodal status and adjuvant trastuzumab were independently associated with RFS, whereas the level of HER-2 amplification nearly reached statistical significance (HR=2.819 (CI 95%, 0.970–8.197), *P*=0.057). Internal validation using bootstrapping confirmed the results only for the pathological nodal status.

There was no significant difference in RFS according to both pCR and level of HER-2 amplification (*P*=0.09, log-rank test). However, the subgroup of HA tumours without pCR had a significantly shorter RFS than did the other subgroups (*P*=0.01, log-rank test; Figure 2C).

### Overall survival according to amplification of HER-2

**Figure 3:**
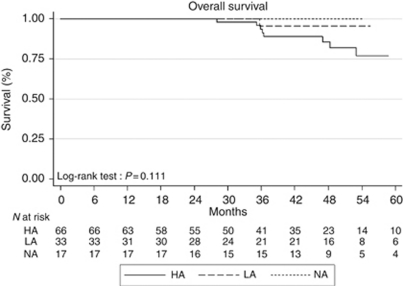
Overall survival according to the HER-2 copy number (Kaplan–Meier estimate). Abbreviations: HA, highly amplified tumours; LA, low-amplified tumours; NA, no amplified tumours.

During follow-up, 11 patients, including 8 with a metastatic recurrence (6 HA tumours, 2 LA tumours), died. There was no significant difference between NA, LA, and HA tumours subgroups for OS (*P*=0.111, log-rank test; Figure 3) or between LA and HA tumours subgroups (*P*=0.22, log-rank test). With Cox univariate analysis, only tumour stage (HR=0.158 (CI 95%, 0.039–0.636), *P*=0.034) and pathological nodal status (HR=7.118 (CI 95%, 1.864–27.177), *P*=0.004) were significantly associated with OS, whereas the level of HER-2 amplification was not (HR=1.974 (CI 95%, 0.413-9.425), *P*=0.394).

## Discussion

Systemic neoadjuvant therapy is the treatment of choice for locally advanced or inflammatory breast cancer. It also facilitates breast conservation in selected patients with operable disease (Kaufmann *et al*, 2006). For patients with HER-2-positive breast cancer (IHC 3+ and/or FISH positive), the addition of trastuzumab to chemotherapy increases pCR rates (Buzdar *et al*, 2005; Gianni *et al*, 2007). We confirm in this larger study our previous results (Arnould *et al*, 2007) regarding the positive correlation between the level of HER-2 amplification determined by FISH and the rate of pCR after trastuzumab-based neoadjuvant therapy. Indeed, we report 55 *vs* 24% (*P*=0.005) of pCR in the subgroup of HA *vs* LA tumours, respectively. In light of these results, the level of HER-2 amplification could be a useful tool to decide whether to administer neoadjuvant therapy and could therefore also increase the rate of conservative surgery. However, this interesting predictive factor needs to be validated in further larger studies. Indeed, our results contrast with those of a smaller series in a neoadjuvant setting, in which response did not correlate with the level of *HER-2* gene amplification measured by FISH (Buzdar *et al*, 2007); this study concerned 45 patients receiving paclitaxel followed by 5-fluorouracile, epirubicin, and cyclophosphamide (FEC) with concurrent trastuzumab. Conversely, our results are consistent with those observed in 57 patients treated with trastuzumab plus chemotherapy (mainly paclitaxel) for a HER-2-positive metastatic breast cancer, in which a clinical objective response was significantly correlated with the level of amplification of HER-2 in FISH (Giuliani *et al*, 2007).

To date there are no data suggesting another mechanism than *HER-2* gene amplification to explain the over-expression of the HER-2 protein. Consequently, both IHC and FISH can be used to determine HER-2 status and the benefit of trastuzumab in breast cancer (Wolff *et al*, 2007). In this study, after centralised analyses, 10 (8%) tumours were subsequently scored IHC 1+ (no pCR was observed in this subgroup, data not shown). This highlights the modest inter-laboratory reproducibility of IHC results. This observation is in line with a recent critical review of the ASCO/CAP guidelines (Sauter *et al*, 2009), which concluded that inherent technical properties strongly argue for primary HER-2 FISH testing. Furthermore, although IHC and FISH have shown high concordance in some studies, reproducibility remains insufficient in others (Paik *et al*, 2002; Roche *et al*, 2002; Mass *et al*, 2005). In our study, 6 out of 11 (55%) tumours with an IHC score of 2+ were finally considered NA with the FISH assay (with only one pCR) and the patient with a 3+ tumour in IHC, which was NA in FISH did not benefit from trastuzumab (data not shown).

Currently, 52 weeks of adjuvant trastuzumab are recommended for the treatment of HER-2-positive breast cancer with a high risk of relapse. This regimen has improved both RFS and OS (Piccart-Gebhart *et al*, 2005; Romond *et al*, 2005; Smith *et al*, 2007; Slamon *et al*, 2009). Several trials in progress are comparing this standard with a shorter exposure to trastuzumab: 9 weeks as in the SOLD study (NCT00593697) and the Short HER study (NCT00629278) or 26 weeks (PHARE study). In a recently published study, a brief course of trastuzumab (9 weeks) administered concomitantly with docetaxel followed by three cycles of FEC tended to improve RFS but not OS, compared with the same regimen without trastuzumab (Joensuu *et al*, 2009). In our study, adjuvant trastuzumab, administered in addition to the 18 pre-operative injections, significantly improved RFS (*P*=0.003). All these results are not in favour of lightening adjuvant trastuzumab. The above mentioned studies should resolve this question.

Pathological complete response is often considered a surrogate marker of outcome after neoadjuvant chemotherapy. Indeed, in several large trials with anthracycline and/or taxanes-based neoadjuvant therapy, RFS and OS rates were significantly improved when pCR had been achieved (Fisher *et al*, 1998; Kuerer *et al*, 1999; Guarneri *et al*, 2006). These studies were performed before the assessment of HER-2 status and the use of trastuzumab. In more recent studies with trastuzumab-based neoadjuvant therapy, the association between pCR and RFS has been inconclusive, sometimes statistically associated (Buzdar *et al*, 2007), but sometimes not (Hurley *et al*, 2006; Shimizu *et al*, 2009) as was the case in our study, despite a long follow-up. This prognostic factor thus remains controversial.

To our knowledge, this is the first study to report the outcome of patients after trastuzumab-based neoadjuvant therapy according to the level of HER-2 amplification in FISH. Although the increase in the number of *HER-2* gene copies had a significant positive impact on the pCR rate, there was no significant difference between HA tumours (>10 *HER-2* gene copies per nuclei) and LA tumours (6–10 *HER-2* gene copies per nuclei) for either RFS or OS after a median follow-up of 46 months. However, RFS tended to be better in the HER-2 LA group (*P*=0.057). A large population-based cohort, treated before the use of trastuzumab, had already shown that OS in breast cancer was not significantly different according to the level of HER-2 amplification, for patients with a HER-2/CEP17 ratio >2.2 (Jensen *et al*, 2008). Since the era of trastuzumab therapy, the link between the level of HER-2 amplification and the outcome of patients has been investigated only in the metastatic and adjuvant settings. A retrospective analysis was performed in 33 patients with HER-2-positive metastatic breast cancer receiving trastuzumab (Gullo *et al*, 2009): patients with a high HER-2/CEP17 ratio had shorter time-to-progression and OS than did those with lower ratios (Gullo *et al*, 2009), although the difference did not reach statistical significance, probably because of the sample size and the relatively short follow-up. Dowsett *et al* (2009) analyzed whether the degree of HER-2 amplification (*HER-2* gene copy number and HER-2/CEP17 ratio) influenced the clinical outcome in patients with HER-2-positive breast cancer randomised in the two HERA trial (Piccart-Gebhart *et al*, 2005) arms with or without 1 year of trastuzumab after adjuvant chemotherapy. Although there was an apparent trend towards shorter disease-free survival with increasing *HER-2* gene copy numbers or increasing HER-2 FISH ratios, the differences were not statistically significant. However, in this study, the median follow-up was only 2 years.

In conclusion, the level of *HER-2* gene amplification using FISH assays significantly influenced pCR but neither RFS nor OS in non-metastatic breast cancer treated with trastuzumab-based neoadjuvant therapy. However, the subgroup of patients with HA tumours (>10 signals per nuclei) tended to have a shorter RFS. This result suggests that a high level of HER-2 amplification could be a poor prognostic factor even though it was associated with a good initial sensitivity to trastuzumab. Further larger and longer studies are needed to confirm this hypothesis.

## Figures and Tables

**Table 1:** Patient and tumour characteristics according to the level of HER-2 amplification

**Characteristic**	**Total (*n*=116)**	**NA (FISH; *n*=17)**	**LA (FISH; *n*=33)**	**HA (FISH; *n*=66)**	***P* (3 groups; *Fisher exact test*)**	***P* (NA *vs* LA/HA; *Fisher exact test*)**	**p (LA *vs* HA) (*Fisher exact test*)**
*Mean age*	46.6	46.6	48.5	45.6			
(Range), year	(26.5–76.4)	(32–62)	(29–76.4)	(26.5–66)			
							
*Tumour stage*					0.699	0.474	0.710
T1	16 (14%)	3 (18%)	4 (12%)	9 (14%)			
T2	73 (63%)	12 (70%)	19 (58%)	42 (64%)			
T3–T4	27 (23%)	2 (12%)	10 (30%)	15 (22%)			
							
*Nodal status*					0.330	0.358	0.363
N0	55 (47%)	11 (65%)	12 (36%)	32 (49%)			
N1	59 (51%)	6 (35%)	20 (61%)	33 (50%)			
N2	2 (2%)	0	1 (3%)	1 (1%)			
							
*Tumour grade*					0.057	0.890	**0.010**
SBR1	5 (4%)	1 (6%)	0	4 (6%)			
SBR2	57 (49%)	9 (53%)	23 (70%)	25 (38%)			
SBR3	47 (41%)	6 (35%)	10 (30%)	31 (47%)			
Unknown	7 (6%)	1 (6%)	0	6 (9%)			
							
*Hormone receptor status*					0.072	**0.017**	0.782
Positive	68 (59%)	13 (76%)	20 (61%)	35 (53%)			
Negative	43 (37%)	2 (12%)	12 (36%)	29 (44%)			
Unknown	5 (4%)	2 (12%)	1 (3%)	2 (3%)			
							
*Neoadjuvant treatment*					0.315^a^	0.145^a^	0.670^a^
TDC	63 (54%)	12 (70%)	18 (55%)	33 (50%)			
TD	53 (46%)	5 (30%)	15 (45%)	33 (50%)			
							
*Central IHC score*					<**0.001**	<**0.001**	**0.003**
1+	10 (8%)	10 (59%)	0	0			
2+	11 (10%)	6 (35%)	5 (15%)	0			
3+	95 (82%)	1 (6%)	28 (85%)	66 (100%)			
							
*Pathological response*					<**0.001**^a^	**0.003** a	**0.004** a
pCR	45 (39%)	1 (6%)	8 (24%)	36 (55%)			
Non-pCR	71 (61%)	16 (94%)	25 (76%)	30 (45%)			
							
*Adjuvant trastuzumab*					0.769	0.557	0.872^a^
No	32 (28%)	6 (35%)	9 (27%)	17 (26%)			
Yes	84 (72%)	11 (65%)	24 (72%)	49 (74%)			

Abbreviations: FISH=fluorescence *in situ* hybridization; HA=highly amplified tumours; IHC=immunohistochemisry; LA=low-amplified tumours; NA=no amplified tumours; non-pCR=absence of complete pathological response; pCR=pathological complete response; SBR=Scarff–Bloom–Richardson; TD=trastuzumab–docetaxel; TDC=trastuzumab–docetaxel–carboplatin.

a *χ*^*2*^-test. Values in bold: *P*<0.05.

**Table 2:** Univariate and multivariate logistic regression for predictive factors of pathological complete response

	**pCR**	**Univariate analysis**			**pCR**	**Multivariate analysis**			**Bootstrapping** a	
	**No/yes**				**No/yes**					
	***N*=99**	**OR**	**95% CI**	***P***	***N*=92**	**OR**	**95% CI**	***P***	**95% CI**	***P***
*Tumour stage*				0.714				0.537		0.691
T1	8/5	1	—		7/4	1				
T2	32/29	1.168	(0.311–4.387)		31/27	1.478	(0.290–7.529)		(0.009–228.220)	
T3–T4	15/10	0.781	(0.166–3.675)		14/9	0.791	(0.125–4.998)		(0.004–162.119)	
										
*Study*				0.570						
GETN(A)-1	28/23	1								
TAXHER-S01	13/7	0.602	(0.408–1.181)							
GFLCC	14/14	1.182	(0.725–1.464)							
										
*Tumour grade*				0.083				0.247		0.534
SBR1	3/1	1	—		3/1	1				
SBR2	32/16	1.476	(0.140–15.613)		31/16	2.407	(0.217–26.645)		(0–35 700 000)	
SBR3	18/23	3.987	(0.361–44.019)		18/23	4.761	(0.423–53.593)		(0–69 800 000)	
Unknown	2/4									
										
*Study*				0.706						
GETN(A)-1	28/23	1								
TAXHER-S01	13/7	1.043	(0.943–1.324)							
GFLCC	14/14	1.523	(0.423–1.544)							
										
*Hormone receptor*				0.299				0.166		0.250
Negative	20/21	1	—		19/21					
Positive	33/22	0.626	(0.271–1.445)		33/19	0.496	(0.184–1.338)		(0.150–1.638)	
Unknown	2/1									
										
*Study*				0.572						
GETN(A)-1	28/23	1								
TAXHER-S01	13/7	0.686	(0.500–1.230)							
GFLCC	14/14	1.302	(0.589–1.500)							
										
*Amplification (FISH)*				**0.005**				**0.024**		0.086
LA	25/8	1	—		24/8	1				
HA	30/36	3.862	(1.508–9.892)		28/32	3.190	(1.162–8.759)		(0.848–12.006)	
										
*Study*				0.520				0.632		0.631
GETN(A)-1	28/23	1			26/21	1				
TAXHER-S01	13/7	0.590	(0.354–1.193)		13/7	0.894	(0.220–3.632)		(0.181–4.412)	
GFLCC	14/14	1.188	(0.727–1.451)		13/12	1.584	(0.533–4.712)		(0.560–4.482)	

Abbreviations: CI=confidence interval; FISH=fluorescence *in situ* hybridisation; GFCLCC=Georges–François Leclerc Cancer Center database; HA=highly amplified tumours; LA=low-amplified tumours; N=number; OR=odds ratio; pCR=pathological complete response; SBR=Scarff–Bloom–Richardson.

a 100 replications. Values in bold: *P*<0.05.

**Table 3:** Univariate and multivariate Cox analysis of predictive factors of recurrence-free survival

	**Recurrence or death**	**Univariate analysis**			**Multivariate analysis**			**Bootstrapping^a^**	
	**No/yes, *N*=99**	**HR**	**95% CI**	***P***	**HR, N=98**	**95% CI**	***P***	**95% CI**	***P***
*Tumour stage*				0.116			0.471		0.978
T1	7/6	1	—		1	—			
T2	47/14	0.446	(0.160–1.241)		0.642	(0.181–2.279)		(0.007–56.910)	
T3–T4	22/3	0.215	(0.048–0.972)		0.344	(0.062–1.911)		(0–6 160 872)	
									
*Tumour grade*				0.260					
SBR1	2/2	1	—						
SBR2	39/9	0.279	(0.058–1.351)						
SBR3	31/10	0.414	(0.082–2.076)						
Unknown	4/2								
									
*Hormone receptor*				0.175					
Negative	28/13	1	—						
Positive	46/9	0.547	(0.229–1.308)						
Unknown	2/1								
									
*Pathological nodal status*				**0.006**			**0.005**		**0.022**
Negative	59/12	1	—		1	—			
Positive	16/11	3.247	(1.396–7.552)		3.928	(1.524–10.127)		(1.219–12.659)	
Unknown	1/0								
									
*Amplification (FISH)*				0.199			0.057		0.883
LA	28/5	1	—		1	—			
HA	48/18	1.918	(0.710–5.180)		2.819	(0.970–8.197)		(0–2 667 202)	
									
*pCR*				0.161					
No	40/15	1	—						
Yes	36/8	0.523	(0.212–1.293)						
									
*pCR and FISH*				0.146					
No+HA	19/11	1	—						
No+LA	21/4	0.385	(0.120–1.228)						
Yes+HA	29/11	0.401	(0.148–1.090)						
Yes+LA	7/1	0.204	(0.025–1.669)						
									
*Adjuvant Trastuzumab*				**0.003**			**0.037**		0.779
No	14/12	1			1				
Yes	62/11	0.157	(0.045–0.539)		0.214	(0.050–0.914)		(0–10 456.280)	
									
Harrell's *C*-statistic					0.7745				
AIC					137				

Abbreviations: AIC=Akaike information criterion; CI=confidence interval; FISH=fluorescence *in situ* hybridisation; HA=highly amplified tumours; HR=hazard ratio; LA=low-amplified tumours; N=number; pCR=pathological complete response; SBR=Scarff–Bloom–Richardson.

a 100 replications. Values in bold: *P*<0.05.

## References

[bib1] Arnould L, Arveux P, Couturier J, Gelly-Marty M, Loustalot C, Ettore F, Sagan C, Antoine M, Penault-Llorca F, Vasseur B, Fumoleau P, Coudert BP (2007) Pathologic complete response to trastuzumab-based neoadjuvant therapy is related to the level of HER-2 amplification. Clin Cancer Res 13: 6404–6409 17975153 10.1158/1078-0432.CCR-06-3022

[bib2] Buzdar AU, Ibrahim NK, Francis D, Booser DJ, Thomas ES, Theriault RL, Pusztai L, Green MC, Arun BK, Giordano SH, Cristofanilli M, Frye DK, Smith TL, Hunt KK, Singletary SE, Sahin AA, Ewer MS, Buchholz TA, Berry D, Hortobagyi GN (2005) Significantly higher pathologic complete remission rate after neoadjuvant therapy with trastuzumab, paclitaxel, and epirubicin chemotherapy: results of a randomized trial in human epidermal growth factor receptor 2-positive operable breast cancer. J Clin Oncol 23: 3676–3685 15738535 10.1200/JCO.2005.07.032

[bib3] Buzdar AU, Valero V, Ibrahim NK, Francis D, Broglio KR, Theriault RL, Pusztai L, Green MC, Singletary SE, Hunt KK, Sahin AA, Esteva F, Symmans WF, Ewer MS, Buchholz TA, Hortobagyi GN (2007) Neoadjuvant therapy with paclitaxel followed by 5-fluorouracil, epirubicin, and cyclophosphamide chemotherapy and concurrent trastuzumab in human epidermal growth factor receptor 2-positive operable breast cancer: an update of the initial randomized study population and data of additional patients treated with the same regimen. Clin Cancer Res 13: 228–233 17200359 10.1158/1078-0432.CCR-06-1345

[bib4] Coudert BP, Arnould L, Moreau L, Chollet P, Weber B, Vanlemmens L, Molucon C, Tubiana N, Causeret S, Misset JL, Feutray S, Mery-Mignard D, Garnier J, Fumoleau P (2006) Pre-operative systemic (neo-adjuvant) therapy with trastuzumab and docetaxel for HER2-overexpressing stage II or III breast cancer: results of a multicenter phase II trial. Ann Oncol 17: 409–414 16332965 10.1093/annonc/mdj096

[bib5] Coudert BP, Largillier R, Arnould L, Chollet P, Campone M, Coeffic D, Priou F, Gligorov J, Martin X, Trillet-Lenoir V, Weber B, Bleuse JP, Vasseur B, Serin D, Namer M (2007) Multicenter phase II trial of neoadjuvant therapy with trastuzumab, docetaxel, and carboplatin for human epidermal growth factor receptor-2-overexpressing stage II or III breast cancer: results of the GETN(A)-1 trial. J Clin Oncol 25: 2678–2684 17515572 10.1200/JCO.2006.09.9994

[bib6] Dowsett M, Procter M, McCaskill-Stevens W, de Azambuja E, Dafni U, Rueschoff J, Jordan B, Dolci S, Abramovitz M, Stoss O, Viale G, Gelber RD, Piccart-Gebhart M, Leyland-Jones B (2009) Disease-free survival according to degree of HER2 amplification for patients treated with adjuvant chemotherapy with or without 1 year of trastuzumab: the HERA Trial. J Clin Oncol 27: 2962–2969 19364966 10.1200/JCO.2008.19.7939 PMC2701645

[bib7] Fisher B, Bryant J, Wolmark N, Mamounas E, Brown A, Fisher ER, Wickerham DL, Begovic M, DeCillis A, Robidoux A, Margolese RG, Cruz Jr AB, Hoehn JL, Lees AW, Dimitrov NV, Bear HD (1998) Effect of preoperative chemotherapy on the outcome of women with operable breast cancer. J Clin Oncol 16: 2672–2685 9704717 10.1200/JCO.1998.16.8.2672

[bib8] Gianni L, Eiermann W, Semiglazov V, Manikhas A, Lluch A, Tjulandin S, Zambetti M, Vazquez F, Byakhow M, Lichinister M, Climent MA, Ciruelos E, Ojeda B, Mansutti M, Bozhok A, Boronio R, Feyereislova A, Barton C, Valagussa P, Baselga J (2007) Neoadjuvant trastuzumab plus doxorubicin, paclitaxel and CMF in locally advanced breast cancer (NOAH trial): feasibility, safety and antitumor effects. In 43th Annual Meeting of American Society of Clinical Oncology Chicago

[bib9] Giuliani R, Durbecq V, Di Leo A, Paesmans M, Larsimont D, Leroy JY, Borms M, Vindevoghel A, Jerusalem G, D’Hondt V, Dirix L, Canon JL, Richard V, Cocquyt V, Majois F, Reginster M, Demol J, Kains JP, Delree P, Keppens C, Sotiriou C, Piccart MJ, Cardoso F (2007) Phosphorylated HER-2 tyrosine kinase and Her-2/neu gene amplification as predictive factors of response to trastuzumab in patients with HER-2 overexpressing metastatic breast cancer (MBC). Eur J Cancer 43: 725–735 17251007 10.1016/j.ejca.2006.11.019

[bib10] Gown AM, Goldstein LC, Barry TS, Kussick SJ, Kandalaft PL, Kim PM, Tse CC (2008) High concordance between immunohistochemistry and fluorescence *in situ* hybridization testing for HER2 status in breast cancer requires a normalized IHC scoring system. Mod Pathol 21: 1271–1277 18487992 10.1038/modpathol.2008.83

[bib11] Guarneri V, Broglio K, Kau SW, Cristofanilli M, Buzdar AU, Valero V, Buchholz T, Meric F, Middleton L, Hortobagyi GN, Gonzalez-Angulo AM (2006) Prognostic value of pathologic complete response after primary chemotherapy in relation to hormone receptor status and other factors. J Clin Oncol 24: 1037–1044 16505422 10.1200/JCO.2005.02.6914

[bib12] Gullo G, Bettio D, Torri V, Masci G, Salvini P, Santoro A (2009) Level of HER2/neu gene amplification as a predictive factor of response to trastuzumab-based therapy in patients with HER2-positive metastatic breast cancer. Invest New Drugs 27: 179–183 18663410 10.1007/s10637-008-9155-y

[bib13] Hurley J, Doliny P, Reis I, Silva O, Gomez-Fernandez C, Velez P, Pauletti G, Powell JE, Pegram MD, Slamon DJ (2006) Docetaxel, cisplatin, and trastuzumab as primary systemic therapy for human epidermal growth factor receptor 2-positive locally advanced breast cancer. J Clin Oncol 24: 1831–1838 16549824 10.1200/JCO.2005.02.8886

[bib14] Jensen KC, Turbin DA, Leung S, Miller MA, Johnson K, Norris B, Hastie T, McKinney S, Nielsen TO, Huntsman DG, Gilks CB, West RB (2008) New cutpoints to identify increased HER2 copy number: analysis of a large, population-based cohort with long-term follow-up. Breast Cancer Res Treat 112: 453–459 18193353 10.1007/s10549-007-9887-y

[bib15] Joensuu H, Bono P, Kataja V, Alanko T, Kokko R, Asola R, Utriainen T, Turpeenniemi-Hujanen T, Jyrkkio S, Moykkynen K, Helle L, Ingalsuo S, Pajunen M, Huusko M, Salminen T, Auvinen P, Leinonen H, Leinonen M, Isola J, Kellokumpu-Lehtinen PL (2009) Fluorouracil, epirubicin, and cyclophosphamide with either docetaxel or vinorelbine, with or without trastuzumab, as adjuvant treatments of breast cancer: final results of the FinHer Trial. J Clin Oncol 27: 5685–5692 19884557 10.1200/JCO.2008.21.4577

[bib16] Joensuu H, Kellokumpu-Lehtinen PL, Bono P, Alanko T, Kataja V, Asola R, Utriainen T, Kokko R, Hemminki A, Tarkkanen M, Turpeenniemi-Hujanen T, Jyrkkio S, Flander M, Helle L, Ingalsuo S, Johansson K, Jaaskelainen AS, Pajunen M, Rauhala M, Kaleva-Kerola J, Salminen T, Leinonen M, Elomaa I, Isola J (2006) Adjuvant docetaxel or vinorelbine with or without trastuzumab for breast cancer. N Engl J Med 354: 809–820 16495393 10.1056/NEJMoa053028

[bib17] Kallioniemi OP, Holli K, Visakorpi T, Koivula T, Helin HH, Isola JJ (1991) Association of c-erbB-2 protein over-expression with high rate of cell proliferation, increased risk of visceral metastasis and poor long-term survival in breast cancer. Int J Cancer 49: 650–655 1682277 10.1002/ijc.2910490504

[bib18] Kaufmann M, Hortobagyi GN, Goldhirsch A, Scholl S, Makris A, Valagussa P, Blohmer JU, Eiermann W, Jackesz R, Jonat W, Lebeau A, Loibl S, Miller W, Seeber S, Semiglazov V, Smith R, Souchon R, Stearns V, Untch M, von Minckwitz G (2006) Recommendations from an international expert panel on the use of neoadjuvant (primary) systemic treatment of operable breast cancer: an update. J Clin Oncol 24: 1940–1949 16622270 10.1200/JCO.2005.02.6187

[bib19] Kuerer HM, Newman LA, Smith TL, Ames FC, Hunt KK, Dhingra K, Theriault RL, Singh G, Binkley SM, Sneige N, Buchholz TA, Ross MI, McNeese MD, Buzdar AU, Hortobagyi GN, Singletary SE (1999) Clinical course of breast cancer patients with complete pathologic primary tumor and axillary lymph node response to doxorubicin-based neoadjuvant chemotherapy. J Clin Oncol 17: 460–469 10080586 10.1200/JCO.1999.17.2.460

[bib20] Leyland-Jones B, Gelmon K, Ayoub JP, Arnold A, Verma S, Dias R, Ghahramani P (2003) Pharmacokinetics, safety, and efficacy of trastuzumab administered every three weeks in combination with paclitaxel. J Clin Oncol 21: 3965–3971 14507946 10.1200/JCO.2003.12.109

[bib21] Marty M, Cognetti F, Maraninchi D, Snyder R, Mauriac L, Tubiana-Hulin M, Chan S, Grimes D, Anton A, Lluch A, Kennedy J, O’Byrne K, Conte P, Green M, Ward C, Mayne K, Extra JM (2005) Randomized phase II trial of the efficacy and safety of trastuzumab combined with docetaxel in patients with human epidermal growth factor receptor 2-positive metastatic breast cancer administered as first-line treatment: the M77001 study group. J Clin Oncol 23: 4265–4274 15911866 10.1200/JCO.2005.04.173

[bib22] Mass RD, Press MF, Anderson S, Cobleigh MA, Vogel CL, Dybdal N, Leiberman G, Slamon DJ (2005) Evaluation of clinical outcomes according to HER2 detection by fluorescence *in situ* hybridization in women with metastatic breast cancer treated with trastuzumab. Clin Breast Cancer 6: 240–246 16137435 10.3816/CBC.2005.n.026

[bib23] Paik S, Bryant J, Tan-Chiu E, Romond E, Hiller W, Park K, Brown A, Yothers G, Anderson S, Smith R, Wickerham DL, Wolmark N (2002) Real-world performance of HER2 testing—National Surgical Adjuvant Breast and Bowel Project experience. J Natl Cancer Inst 94: 852–854 12048273 10.1093/jnci/94.11.852

[bib24] Piccart-Gebhart MJ, Procter M, Leyland-Jones B, Goldhirsch A, Untch M, Smith I, Gianni L, Baselga J, Bell R, Jackisch C, Cameron D, Dowsett M, Barrios CH, Steger G, Huang CS, Andersson M, Inbar M, Lichinitser M, Lang I, Nitz U, Iwata H, Thomssen C, Lohrisch C, Suter TM, Ruschoff J, Suto T, Greatorex V, Ward C, Straehle C, McFadden E, Dolci MS, Gelber RD (2005) Trastuzumab after adjuvant chemotherapy in HER2-positive breast cancer. N Engl J Med 353: 1659–1672 16236737 10.1056/NEJMoa052306

[bib25] Press MF, Bernstein L, Thomas PA, Meisner LF, Zhou JY, Ma Y, Hung G, Robinson RA, Harris C, El-Naggar A, Slamon DJ, Phillips RN, Ross JS, Wolman SR, Flom KJ (1997) HER-2/neu gene amplification characterized by fluorescence *in situ* hybridization: poor prognosis in node-negative breast carcinomas. J Clin Oncol 15: 2894–2904 9256133 10.1200/JCO.1997.15.8.2894

[bib26] Roche PC, Suman VJ, Jenkins RB, Davidson NE, Martino S, Kaufman PA, Addo FK, Murphy B, Ingle JN, Perez EA (2002) Concordance between local and central laboratory HER2 testing in the breast intergroup trial N9831. J Natl Cancer Inst 94: 855–857 12048274 10.1093/jnci/94.11.855

[bib27] Romond EH, Perez EA, Bryant J, Suman VJ, Geyer Jr CE, Davidson NE, Tan-Chiu E, Martino S, Paik S, Kaufman PA, Swain SM, Pisansky TM, Fehrenbacher L, Kutteh LA, Vogel VG, Visscher DW, Yothers G, Jenkins RB, Brown AM, Dakhil SR, Mamounas EP, Lingle WL, Klein PM, Ingle JN, Wolmark N (2005) Trastuzumab plus adjuvant chemotherapy for operable HER2-positive breast cancer. N Engl J Med 353: 1673–1684 16236738 10.1056/NEJMoa052122

[bib28] Sauter G, Lee J, Bartlett JM, Slamon DJ, Press MF (2009) Guidelines for human epidermal growth factor receptor 2 testing: biologic and methodologic considerations. J Clin Oncol 27: 1323–1333 19204209 10.1200/JCO.2007.14.8197

[bib29] Shimizu C, Masuda N, Yoshimura K, Tsuda H, Mano M, Ando M, Tamura K, Fujiwara Y (2009) Long-term outcome and pattern of relapse after neoadjuvant chemotherapy in patients with human epidermal growth factor receptor 2-positive primary breast cancer. Jpn J Clin Oncol 39: 484–490 19477897 10.1093/jjco/hyp052

[bib30] Slamon D, Eiermann W, Robert N, Pienkowski T, Martin M, Rolski J, Chan A, Mackey J, Liu M, Pinter T, Valero V, Falkson C, Fornander T, Shiftan T, Olsen S, Buyse M, Kiskartalyi T, Landreau V, Wilson V, Press M, Crown J (2009) Phase III randomized trial comparing doxorubicin and cyclophosphamide followed by docetaxel with doxorubicin and cyclophosphamide followed by docetaxel and trastuzumab with docetaxel, carboplatin and trastuzumab in Her2neu positive early breast cancer patients: BCIRG 006 Study. Cancer Res 69: 500s

[bib31] Slamon DJ, Clark GM, Wong SG, Levin WJ, Ullrich A, McGuire WL (1987) Human breast cancer: correlation of relapse and survival with amplification of the HER-2/neu oncogene. Science 235: 177–182 3798106 10.1126/science.3798106

[bib32] Slamon DJ, Leyland-Jones B, Shak S, Fuchs H, Paton V, Bajamonde A, Fleming T, Eiermann W, Wolter J, Pegram M, Baselga J, Norton L (2001) Use of chemotherapy plus a monoclonal antibody against HER2 for metastatic breast cancer that overexpresses HER2. N Engl J Med 344: 783–792 11248153 10.1056/NEJM200103153441101

[bib33] Smith I, Procter M, Gelber RD, Guillaume S, Feyereislova A, Dowsett M, Goldhirsch A, Untch M, Mariani G, Baselga J, Kaufmann M, Cameron D, Bell R, Bergh J, Coleman R, Wardley A, Harbeck N, Lopez RI, Mallmann P, Gelmon K, Wilcken N, Wist E, Sanchez Rovira P, Piccart-Gebhart MJ (2007) 2-Year follow-up of trastuzumab after adjuvant chemotherapy in HER2-positive breast cancer: a randomised controlled trial. Lancet 369: 29–36 17208639 10.1016/S0140-6736(07)60028-2

[bib34] Van Pelt AE, Mohsin S, Elledge RM, Hilsenbeck SG, Gutierrez MC, Lucci Jr A, Kalidas M, Granchi T, Scott BG, Allred DC, Chang JC (2003) Neoadjuvant trastuzumab and docetaxel in breast cancer: preliminary results. Clin Breast Cancer 4: 348–353 14715110 10.3816/cbc.2003.n.040

[bib35] Wolff AC, Hammond ME, Schwartz JN, Hagerty KL, Allred DC, Cote RJ, Dowsett M, Fitzgibbons PL, Hanna WM, Langer A, McShane LM, Paik S, Pegram MD, Perez EA, Press MF, Rhodes A, Sturgeon C, Taube SE, Tubbs R, Vance GH, van de Vijver M, Wheeler TM, Hayes DF (2007) American Society of Clinical Oncology/College of American Pathologists guideline recommendations for human epidermal growth factor receptor 2 testing in breast cancer. J Clin Oncol 25: 118–145 17159189 10.1200/JCO.2006.09.2775

[bib36] Yamauchi H, Stearns V, Hayes DF (2001) When is a tumor marker ready for prime time? A case study of c-erbB-2 as a predictive factor in breast cancer. J Clin Oncol 19: 2334–2356 11304787 10.1200/JCO.2001.19.8.2334

